# Supplementary figures and images for: A sixfold urban design framework to assess climate resilience: Generative transformation in Negril, Jamaica
Source: PLoS One. 2023 Jun 23;18(6):e0287364. doi: 10.1371/journal.pone.0287364 (PMC10289356; doi:10.1371/journal.pone.0287364)

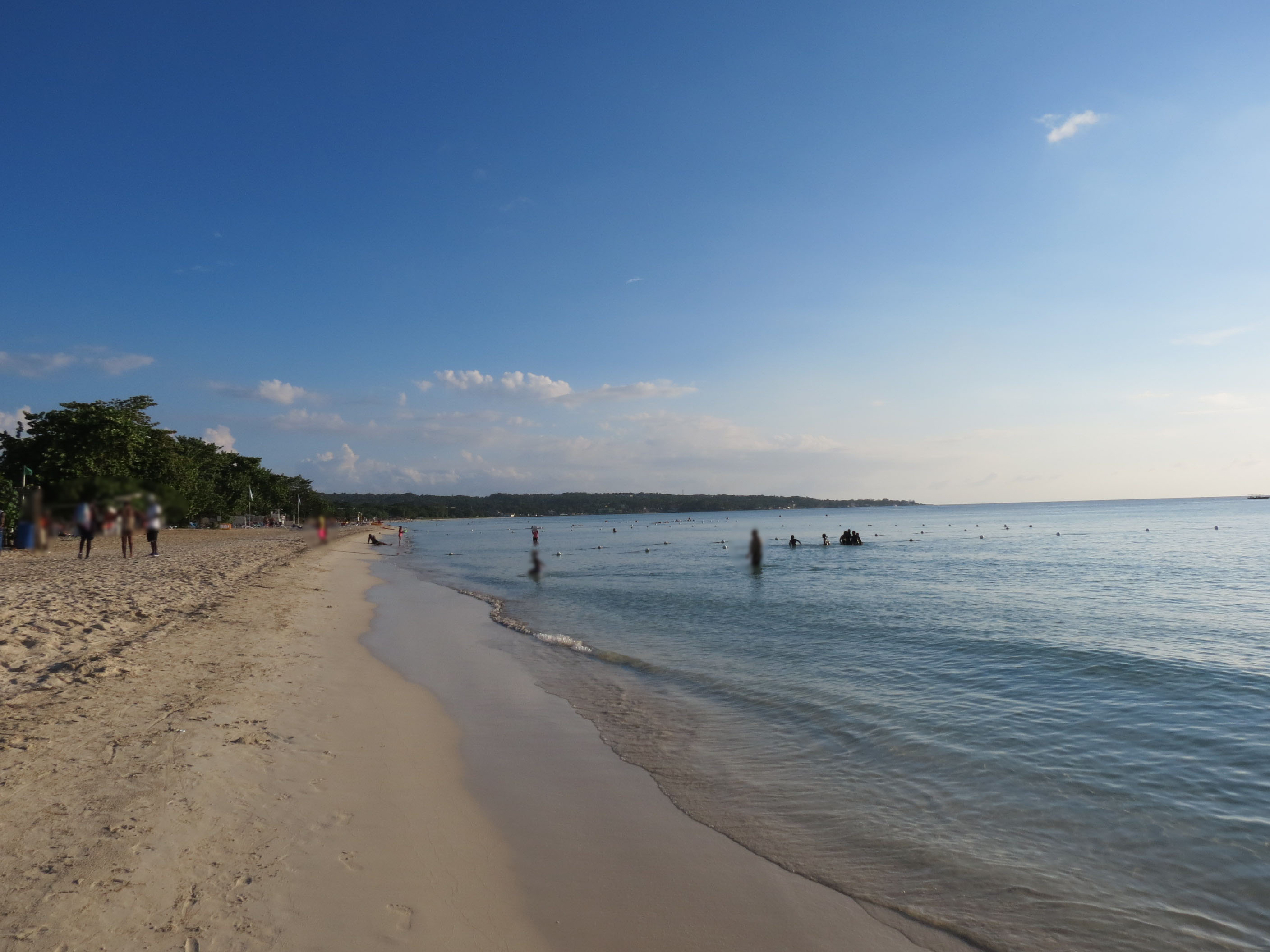

Supplement: S1 File — (ZIP) [file pone.0287364.s001.zip › Archive/IMG_3723.JPG]

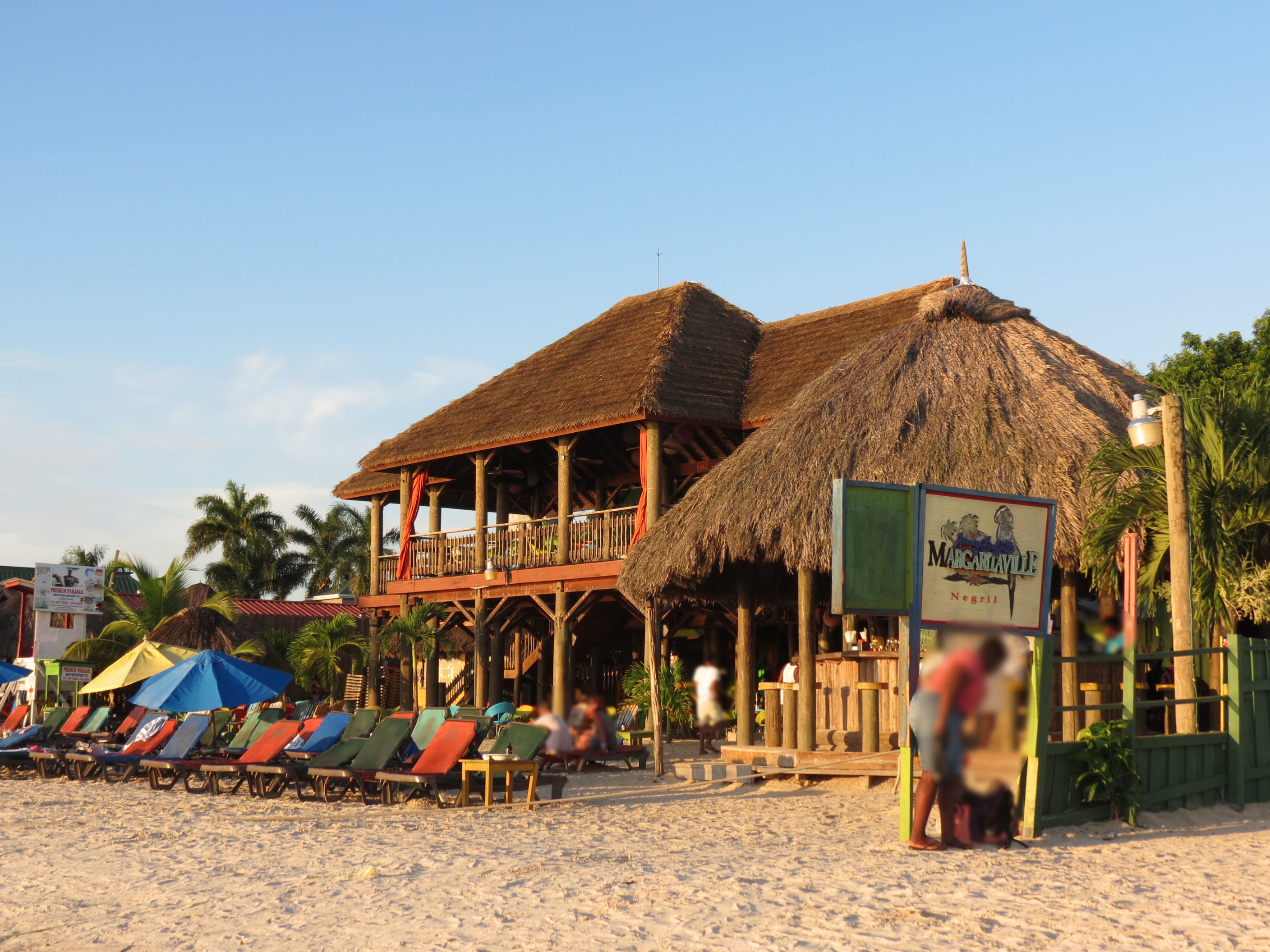

Supplement: S1 File — (ZIP) [file pone.0287364.s001.zip › Archive/IMG_3737.JPG]

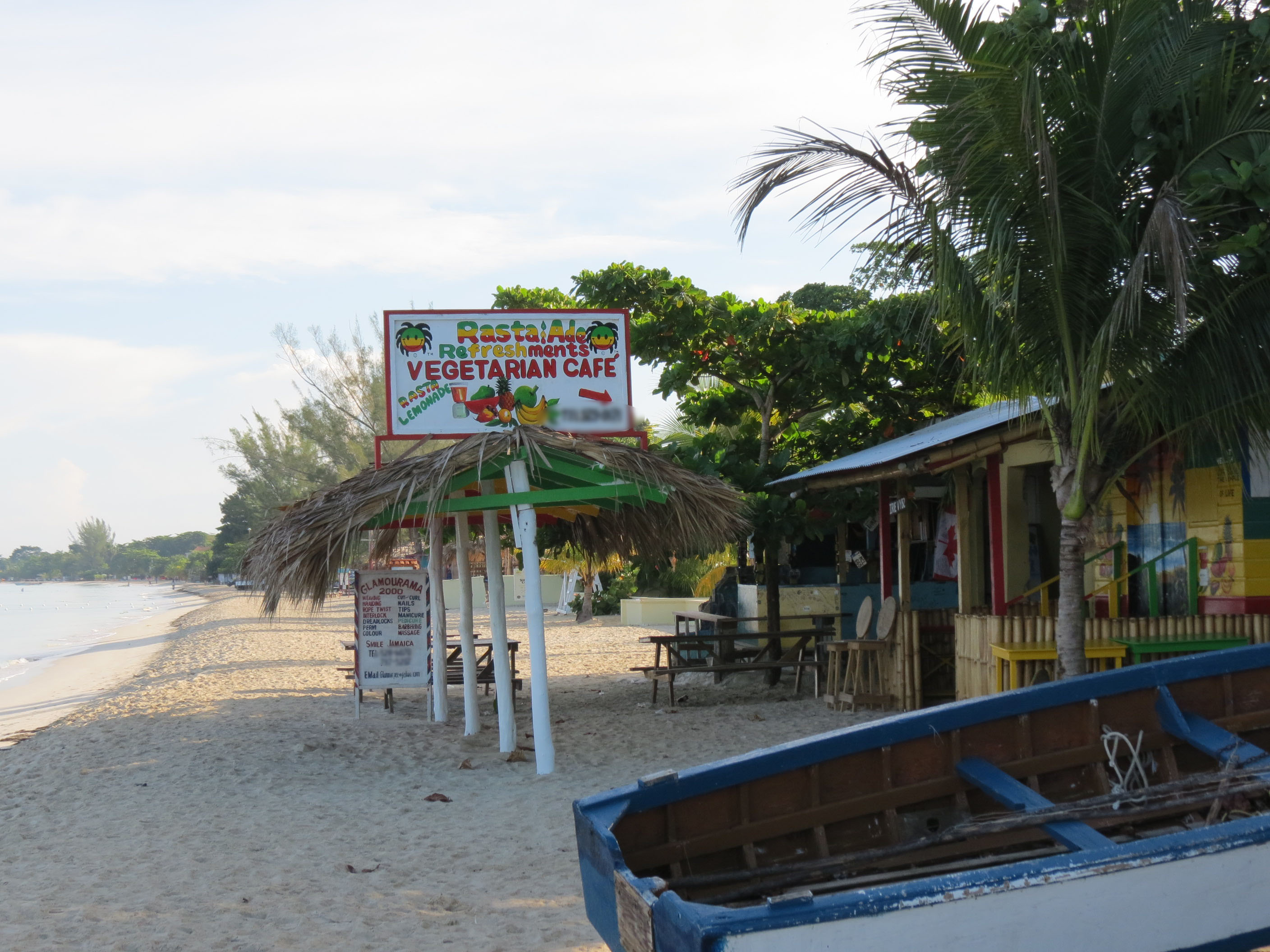

Supplement: S1 File — (ZIP) [file pone.0287364.s001.zip › Archive/IMG_3696.JPG]

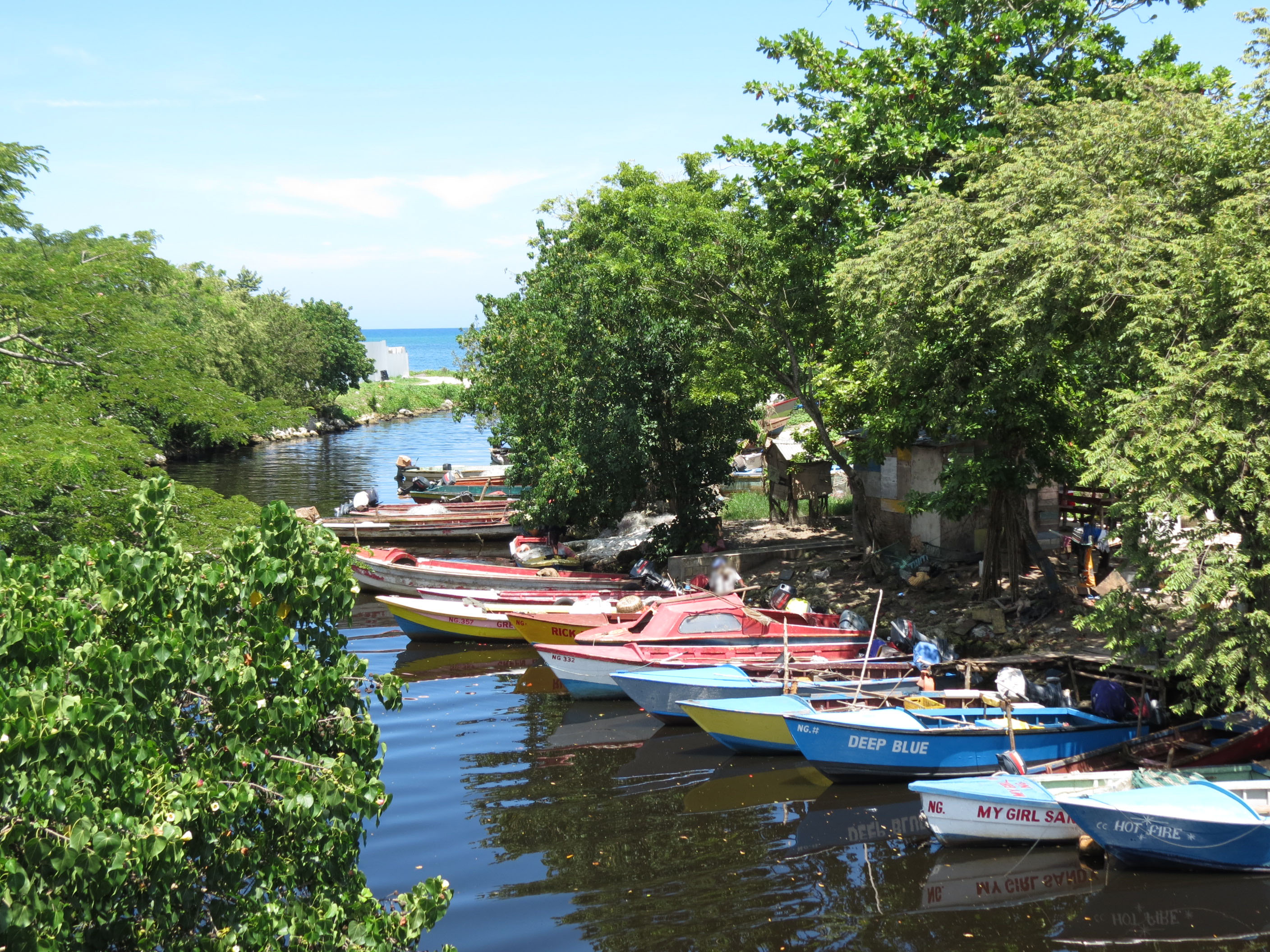

Supplement: S1 File — (ZIP) [file pone.0287364.s001.zip › Archive/IMG_3721.JPG]

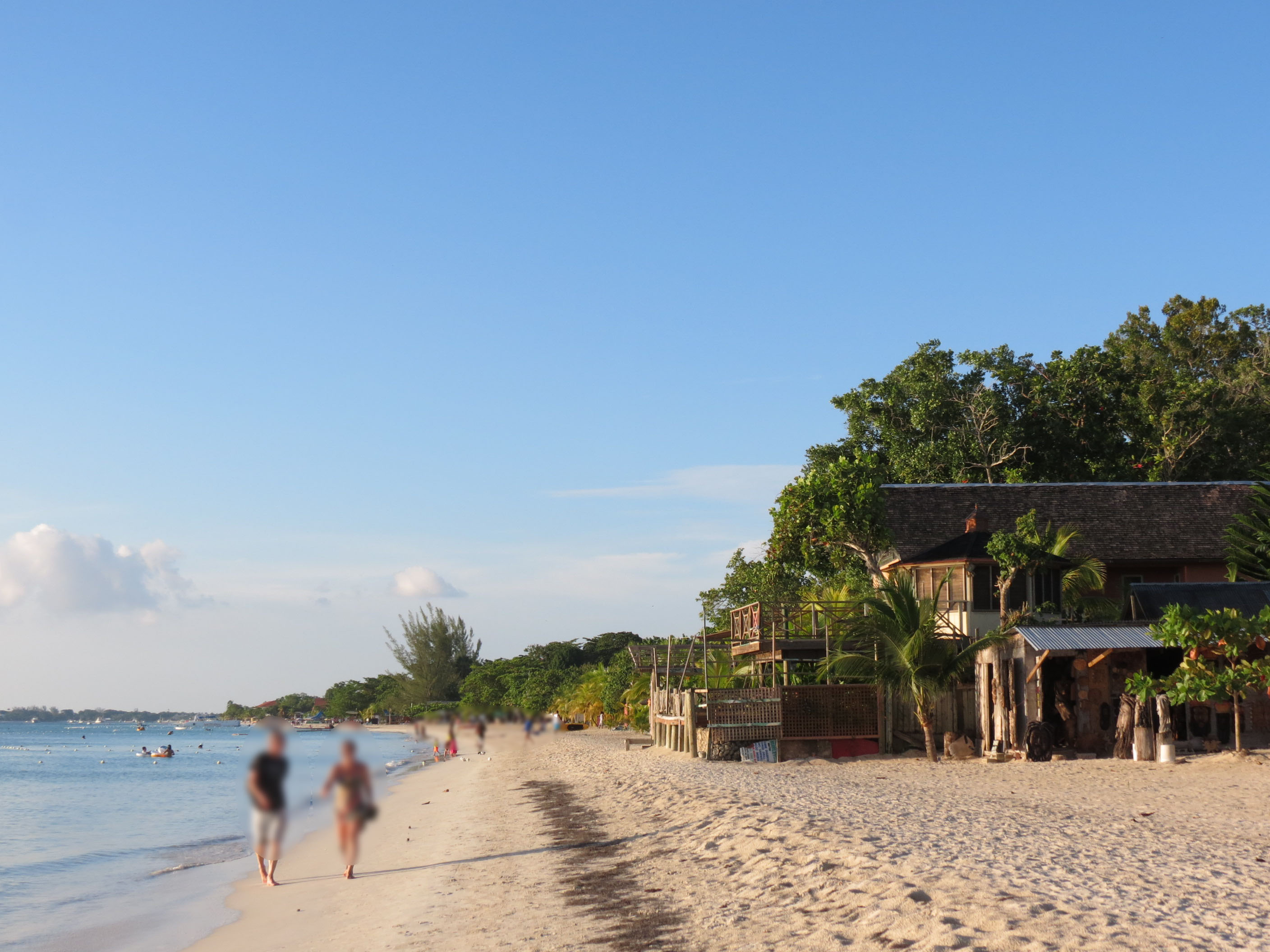

Supplement: S1 File — (ZIP) [file pone.0287364.s001.zip › Archive/IMG_3726.JPG]

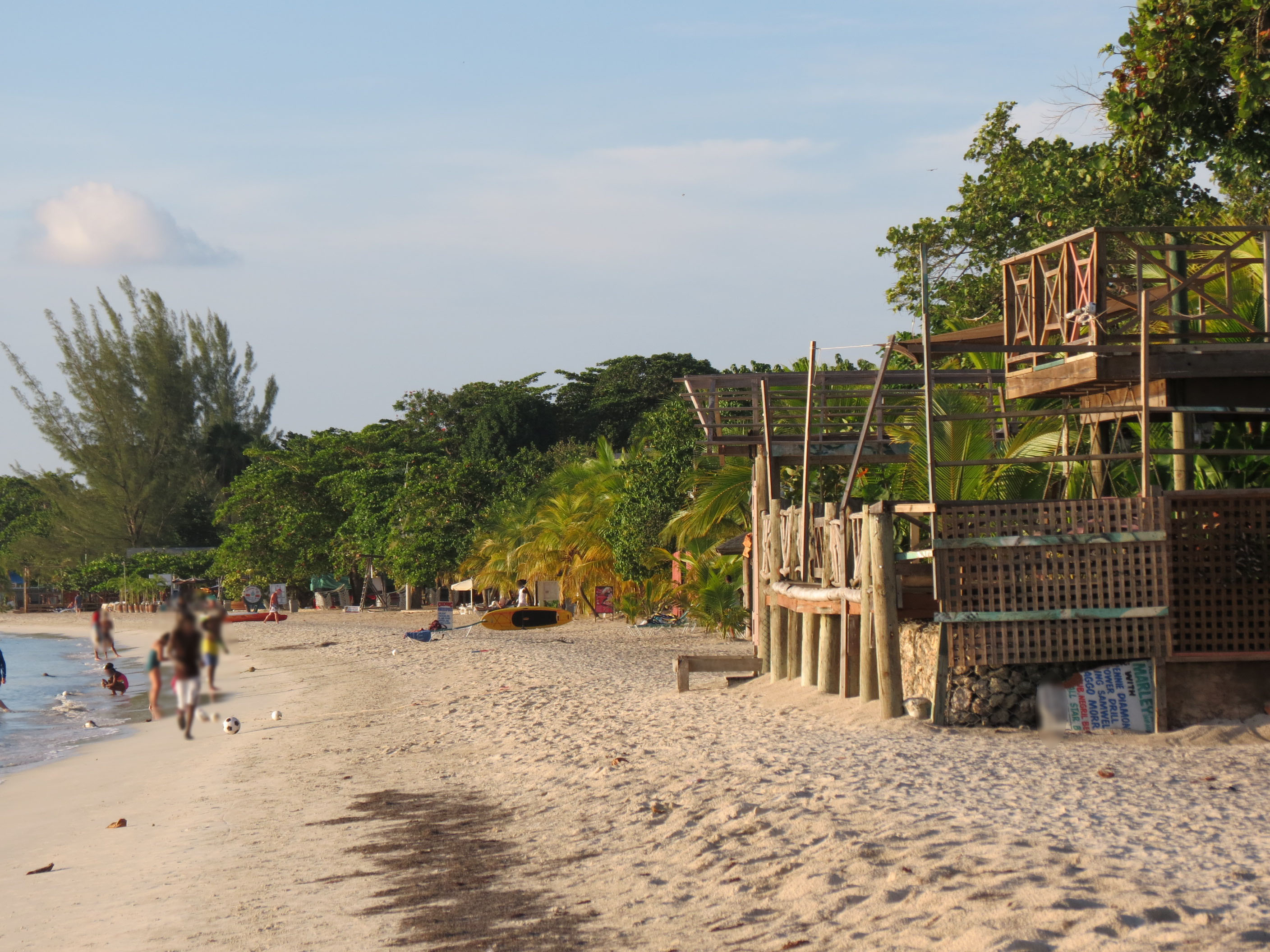

Supplement: S1 File — (ZIP) [file pone.0287364.s001.zip › Archive/IMG_3727.JPG]

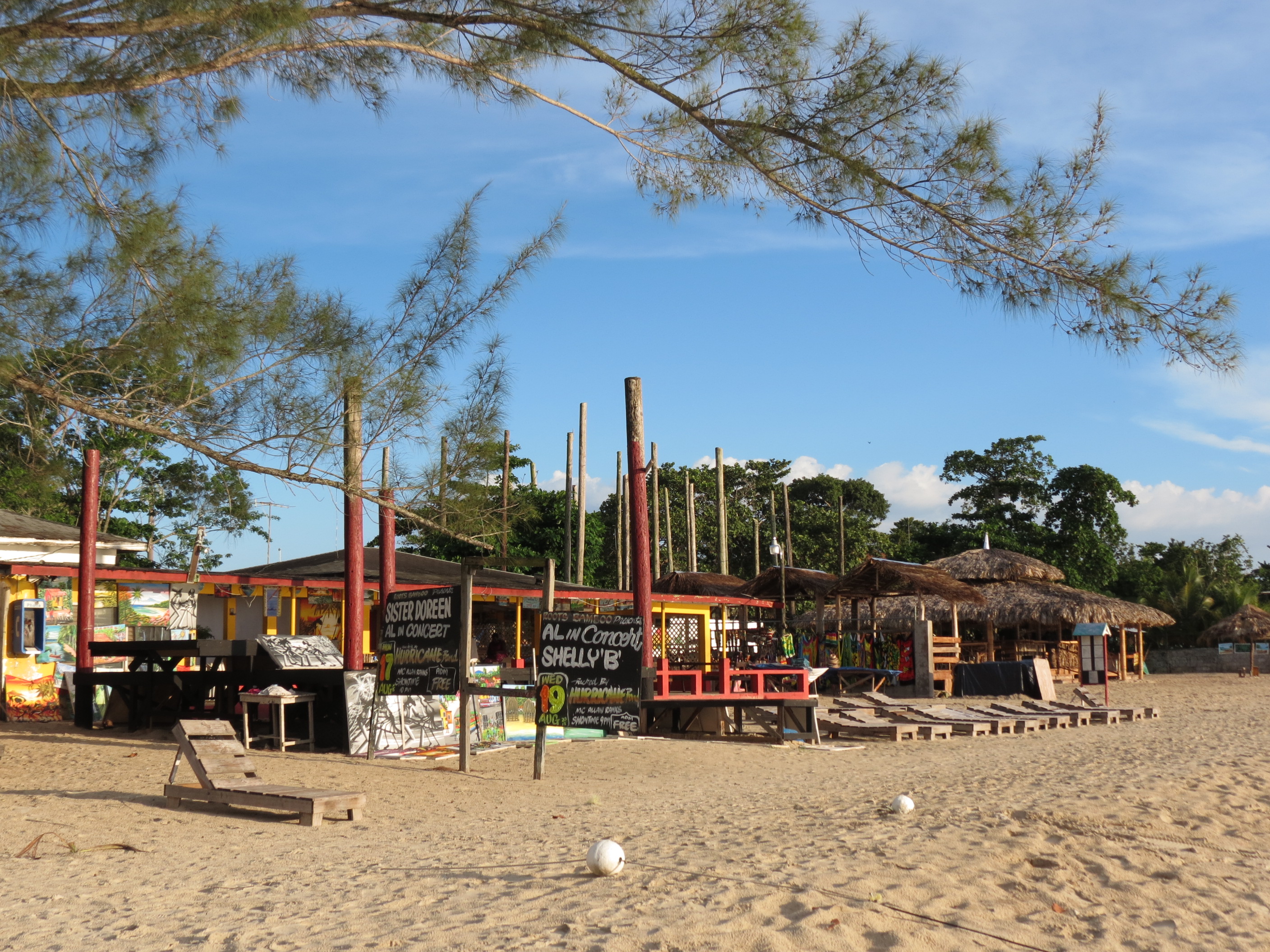

Supplement: S1 File — (ZIP) [file pone.0287364.s001.zip › Archive/IMG_3783.JPG]

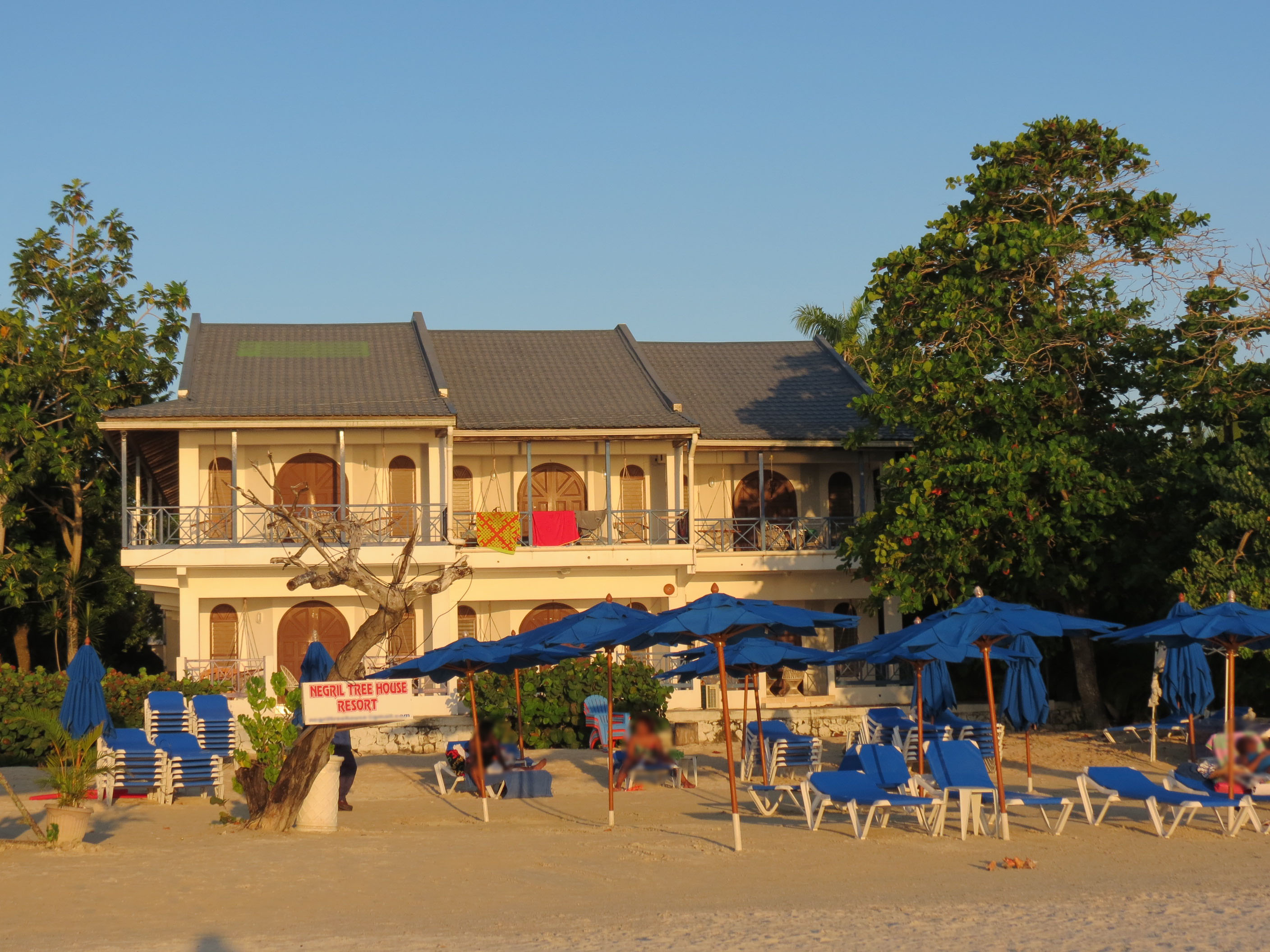

Supplement: S1 File — (ZIP) [file pone.0287364.s001.zip › Archive/IMG_3741.JPG]

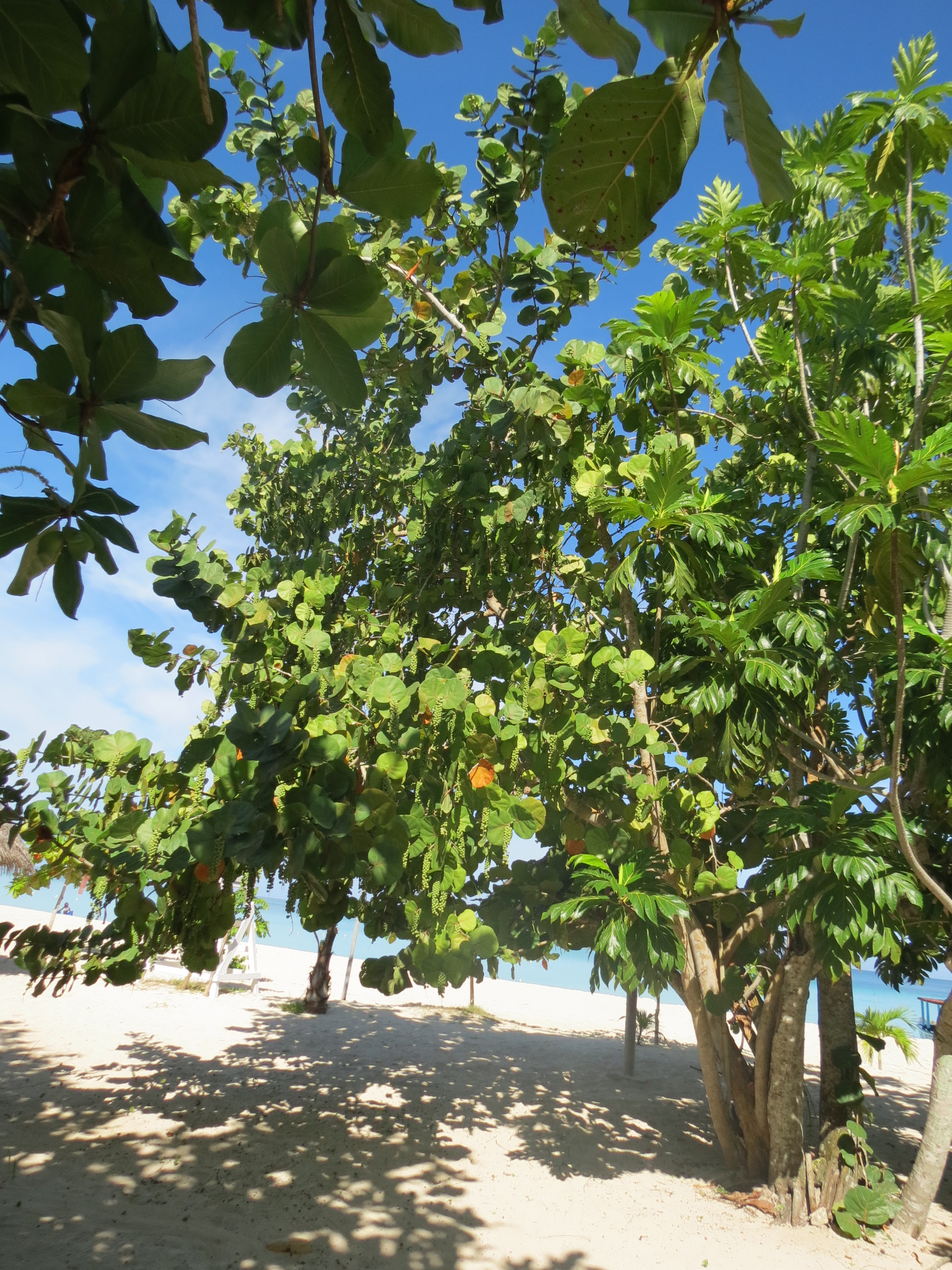

Supplement: S1 File — (ZIP) [file pone.0287364.s001.zip › Archive/IMG_3838.JPG]

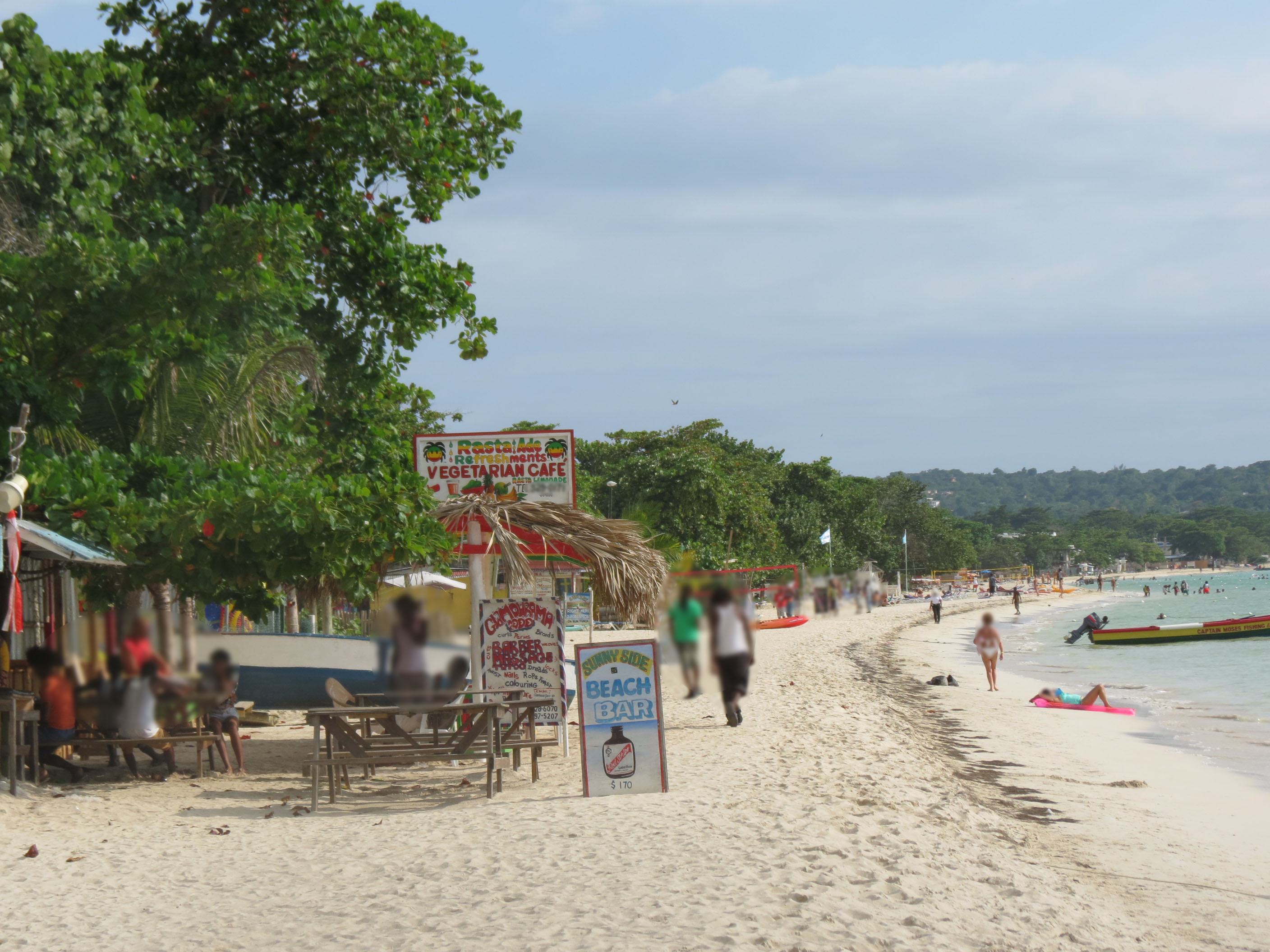

Supplement: S1 File — (ZIP) [file pone.0287364.s001.zip › Archive/IMG_3637.JPG]

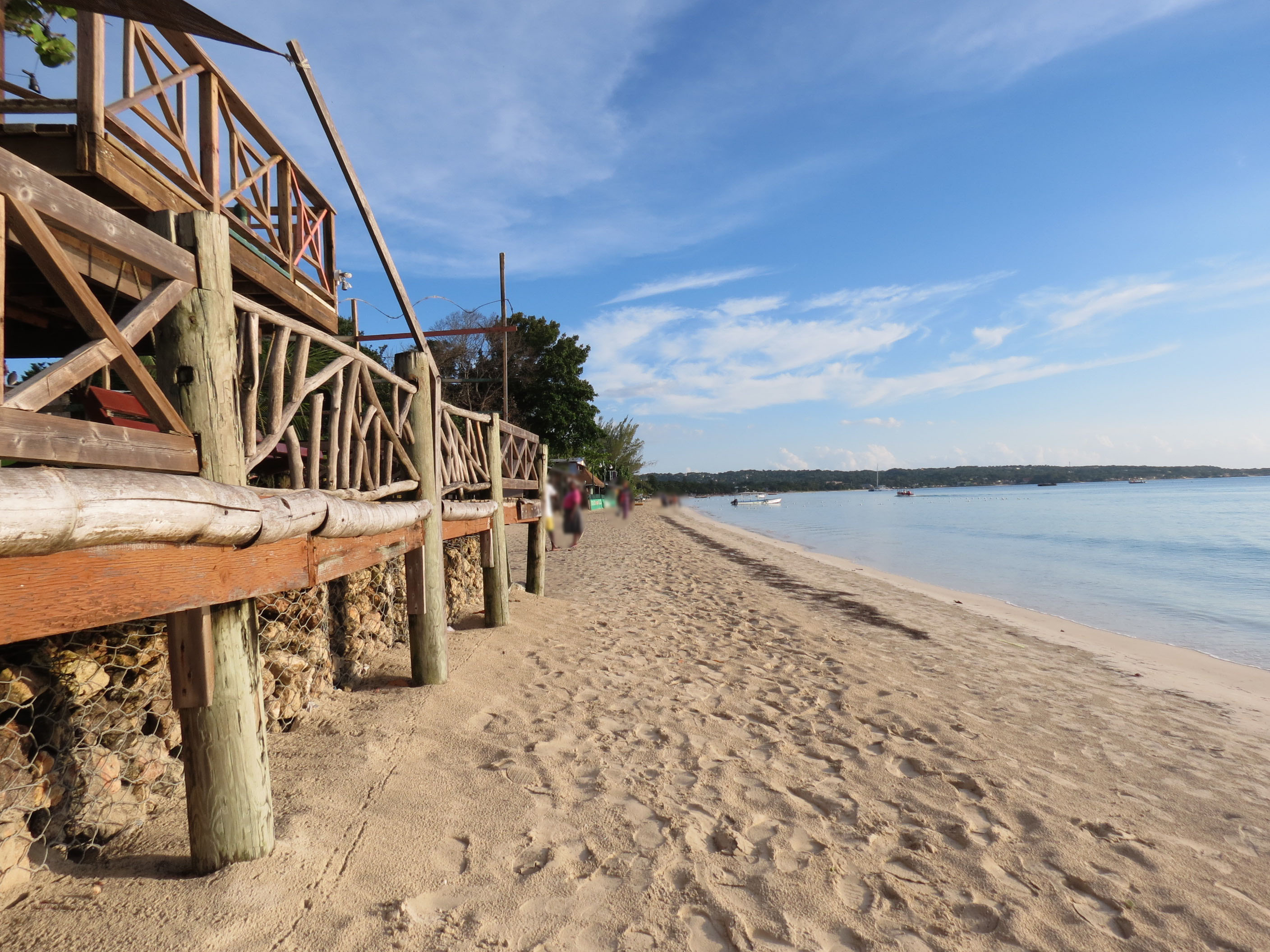

Supplement: S1 File — (ZIP) [file pone.0287364.s001.zip › Archive/IMG_3774.JPG]

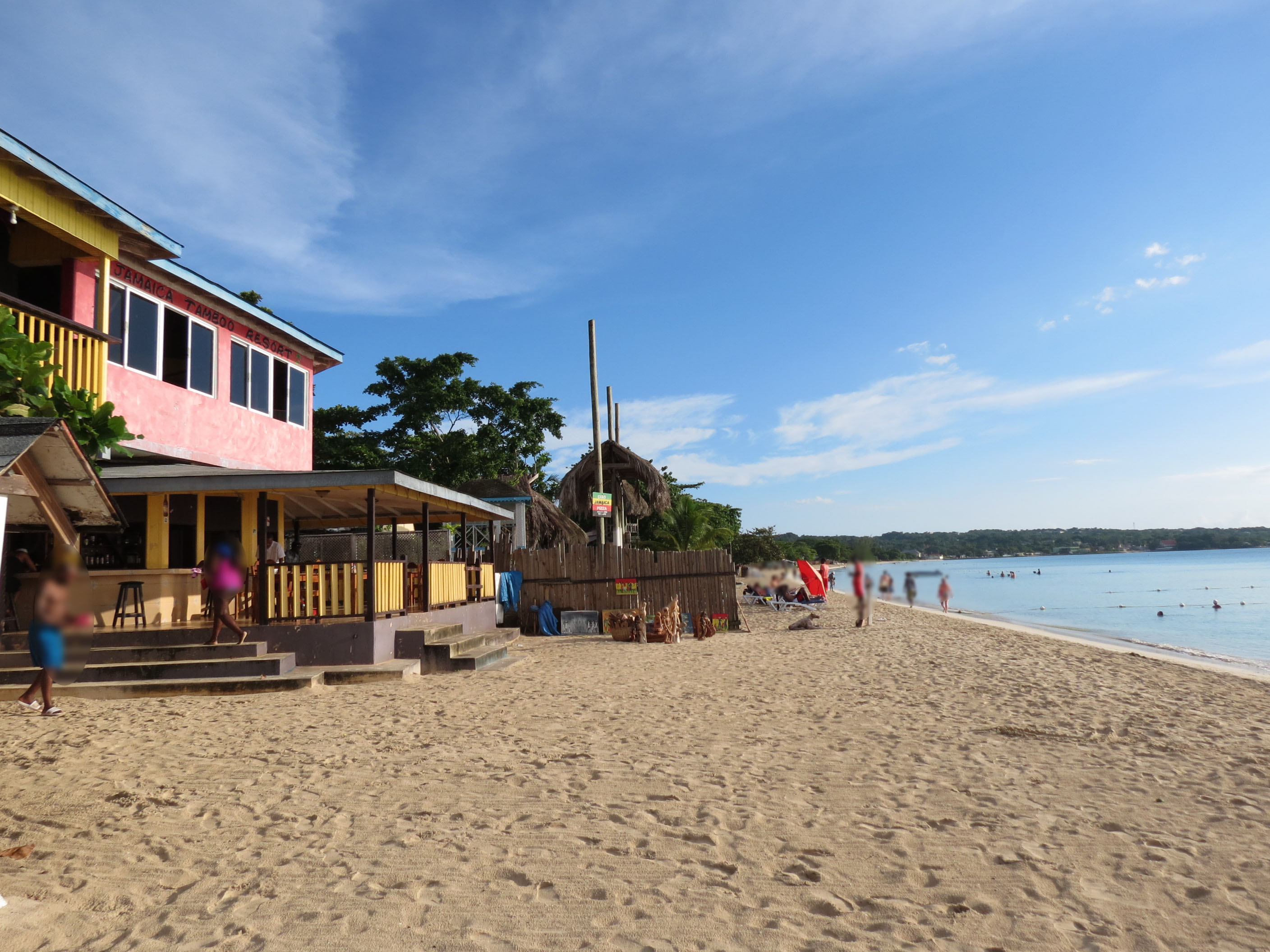

Supplement: S1 File — (ZIP) [file pone.0287364.s001.zip › Archive/IMG_3763.JPG]

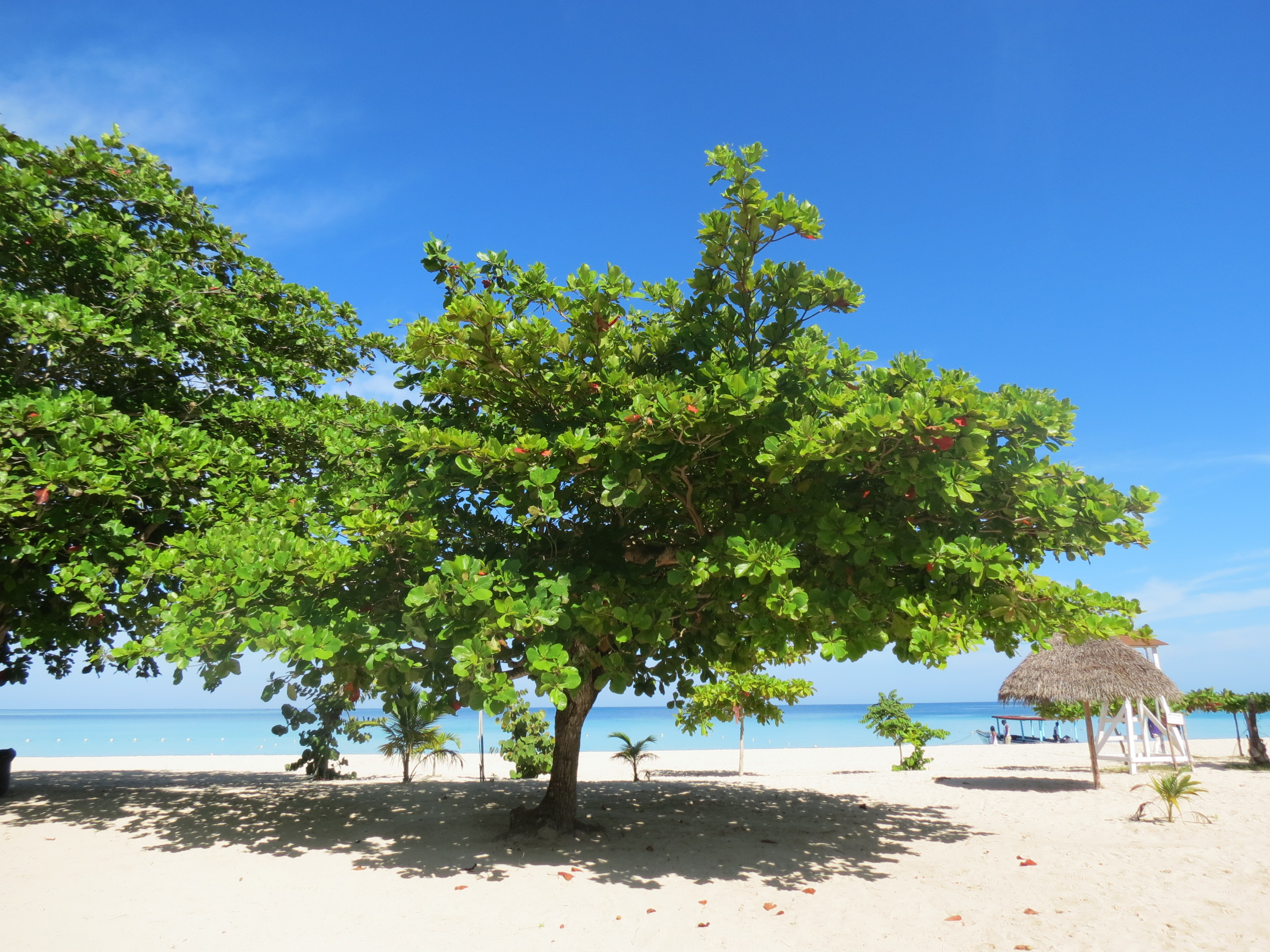

Supplement: S1 File — (ZIP) [file pone.0287364.s001.zip › Archive/IMG_3836.JPG]

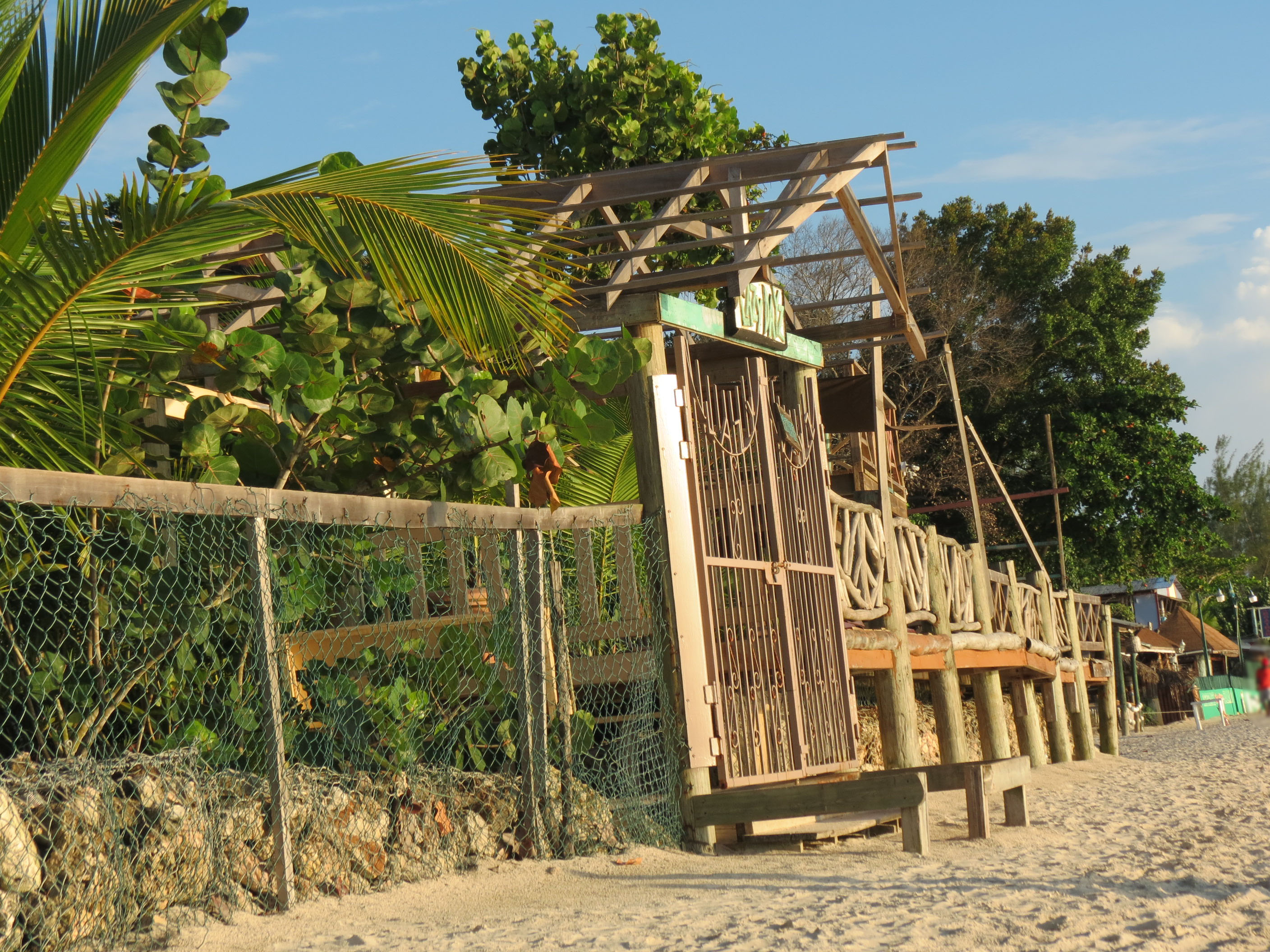

Supplement: S1 File — (ZIP) [file pone.0287364.s001.zip › Archive/IMG_3728.JPG]
