# Supplementary material for: A sixfold urban design framework to assess climate resilience: Generative transformation in Negril, Jamaica
Source: PLoS One. 2023 Jun 23;18(6):e0287364. doi: 10.1371/journal.pone.0287364 (PMC10289356; doi:10.1371/journal.pone.0287364)
Supplement: S1 Appendix — (DOCX) [file pone.0287364.s003.docx]

Appendix A: *Indicators and variables to assess a community’s resilience: a review*

| **Frameworks** | **Indicators** | **Variables** |
| --- | --- | --- |
| Integration with nature  [1, 2] | Wetland (increase/loss) | Increase or loss of land area over time |
|  | Erosion and land degradation | Annual average rate |
|  | Porosity of built infrastructure | Percentage of impervious surface |
|  | Biodiversity | Extent of local species |
|  | Ecosystem services | Extent of preserved ecosystems |
| Social wellbeing  [3-5] | Demographic profiles | Population − number, age, health, gender, education, among others. |
|  | Social capital and networks | Community value-cohesion  Faith-based organization |
|  | Equity | Access to transportation, resources, and services  (health care, education, … etc.) |
|  | Livelihood protection | Amounts of livestock and working animals, tools, and seeds |
|  | Cultural protection | Cultural and heritage resources and religious sites |
| Economic strength  [1, 3] | Employment and assets | Household income, single sector dependency,  property values, and business sizes |
|  | Community disaster reduction fund | Amount of resources and accessibility to them  Financial protection, subsidies, and insurance availability  Public-private partnerships |
| Institutional capacity  [1, 6-8] | Institutional networks | Degrees of collaboration and coordination at  different layers |
|  | Disaster management | Progress of risk assessment, mapping, management, and preparedness plans |
|  | Enforcement capacity | Application of zoning and building regulations |
|  | Governance and local competence | Political fragmenting  Involvement of communities in disaster management  process  Knowledge dissemination and management  Early warning  Local understanding of risk and preparedness |
| Physical infrastructure  [7, 8] | Protection strategies | Number of defence structures (e.g., in coastal areas) |
|  | Lifelines and critical  infrastructure | Age and quality of infrastructure (electricity, water  supply, sanitation, and solid waste disposal) |
|  | Transportation networks | Accessibility of roads  Connectivity of internal road network  Quick and safe evacuation |
|  | Residential, commercial, and  manufacturing establishments | Establishments’ age, stock, and availability  Construction and design safety |

**References used:**

1. Plummer, R. and D. Armitage, *A Resilience-based Framework for Evaluating Aadaptive Co-management: Linking Ecology, Economics and Society in a Complex World.* Ecological Economics, 2007. **61**(1): p. 62-74.

2. Cutter, S.L., et al., *A Place-based Model for Understanding Community Resilience to Natural Disasters.* Global Environmental Change, 2008. **18**(4): p. 598-606.

3. UNISDR, *Sendai Framework for Disaster Risk Reduction 2015 - 2030*. 2015, The United Nations Office for Disaster Reduction

4. Jha, a.K., T.W. Miner, and Z. Stanton-Geddes, eds. *Building Urban Resilience: Principles, Tools, and Practice* 2013, The World Bank Washington, DC.

5. Cutter, S.L., C.G. Burton, and C.T. Emrich, *Disaster Resilience Indicators for Benchmarking Baseline Conditions.* Journal of Homeland Security and Emergency Management, 2010. **7**(1): p. 1-22.

6. ADPC, *Critical Guidelines: Community-based Disaster Risk Management*. 2006, Asian Disaster Preparedness Center: Bangkok.

7. Shaw, R. and I. Team, *Climate Disaster Resilience: Focus on Coastal Urban Cities in Asia.* Asian Journal of Environment and Disaster Management, 2009. **1**: p. 101-116.

8. Joerin, J., et al., *The Adoption of a Climate Disaster Resilience Index in Chennai, India.* Disasters, 2014. **38**(3): p. 540-561.
