# Supplementary material for: A sixfold urban design framework to assess climate resilience: Generative transformation in Negril, Jamaica
Source: PLoS One. 2023 Jun 23;18(6):e0287364. doi: 10.1371/journal.pone.0287364 (PMC10289356; doi:10.1371/journal.pone.0287364)
Supplement: Attachment — Submitted filename: PONE-D-21-17612Review1.docx [file pone.0287364.s004.docx]

PEER REVIEW Manuscript Number: PONE-D-21-17612

Article Type: Research Article

Full Title: A sixfold urban design framework to assess climate resilience: generative

transformation in Negril, Jamaica

Short Title: A sixfold urban design framework to assess climate resilience

The paper is focused in a very important issue as climate resilience is. The author proposes a framework for asses this climate resilience based, essentially, on urban design. Although the six points discussed in the framework are important, I think that, obviously, they are not enough to face all the problems related to climate change. For this reason, I think that a clearer discussion about the scope will be welcomed.

The literature review is wide but as the paper is mixing different areas, the final position remains unclear. In other words, is there other frameworks for assessing climate resilience? Could be better to focus on frameworks or in urban design?. I would appreciate a discussion on specific issue of the paper more than a general discussion on general models. In my case, the final idea of the discussion remains unclear. As the author said, I was expecting to limit the intersection between urban design and resilience, and to give specific actions for being done.

In fact, the sixfold framework comes from six urban design concepts, but it is not enough discussed if is the main paper contribution. The emergence of the six concepts should be better explained and to specify why these six concepts.

Having frameworks helps to give order to assessing methods but, in general, the application of indicators and measures is the path. In this case, the author present indicators and variables but it is not possible to apply them to case study.

Perhaps, storytelling needs to be rethought and strengthening the links between the framework and the application. In any case, the work done with all the interviews is very valuable and should be appreciated by the politicians and public servants of the case study.

I was expecting new ideas and new ways to adapt to climate change in the case of increasing sea-level and it was not the case. In fact, economic and political barriers appear, as it is usual.

Results are very interesting for decision-making but the author is proposing actions that should be part of a resilience plan.

For this reason, I consider that although the paper is interesting, it is unsuitable for publication in its present form. Nevertheless, the study shows sufficient potential to encourage the author to resubmit a revised version focusing on the main contribution to the academic community. In other case, the paper is nearer to a policy practice than an academic paper.
