# Supplementary material for: A sixfold urban design framework to assess climate resilience: Generative transformation in Negril, Jamaica
Source: PLoS One. 2023 Jun 23;18(6):e0287364. doi: 10.1371/journal.pone.0287364 (PMC10289356; doi:10.1371/journal.pone.0287364)
Supplement: Attachment — Submitted filename: Response to Reviewers comments_PONE-D-21-17612.docx [file pone.0287364.s005.docx]

**Response to Reviewers**

**Reviewer 1**

The topic is very interesting and timely and developing methods for assessing resilience to climate change is much needed. However, I am recommending major revisions as there are several major aspects of the paper that need revision. My first major comment is that the writing style can be clunky and difficult to follow at times. For example, the author(s) use long paragraphs, long sentences, commas, and semi colons, instead of shorter paragraphs and sentences to break apart separate concepts and ideas. I suggest the authors thoroughly read through and revise the manuscript to address this issue and also try to be more concise throughout.

*Authors’ response:*

We thank Reviewer 1 for the constructive and detailed comments and for appreciating our efforts. As advised, we have revised the entire manuscript to improve the writing style.

Second, for readers without an urban design or planning background, the author(s) need to better define and explain some of the terminology. This manuscript should be more accessible to a general audience.

*Authors’ response:*

Thank you again for this constructive feedback. We have defined the key terminologies, namely: urban form and morphology, in the texts and in the endnote.

Third, in the introduction and background sections, as well as the discussion section, the writing is very much kept at the theoretical level. I think it would be improved if specific examples, contexts, and case studies were integrated into these sections. It would make the ideas and arguments more concrete. As well as compare the Jamaica case with other similar cases.

*Authors’ response:*

We thank the reviewer for this critical observation. We have integrated examples (marked with Cyan) into the sections to relate our theoretical contents to specific examples from case studies. We also added a few cases to the discussion section to compare our findings from Jamaica.

Fourth, the entire manuscript should be revised to be more concise and direct. At times the text strays or appears rambling instead of staying on point and being direct.

Lastly, I suggest that the author(s) integrate to better integrate the various data sources. In the results section, they mostly present the interview data, and while that it interested, it would be nicely complemented by some of the secondary data, including maps.

*Authors’ response:*

We agree with Reviewer 1’s comments. We have revised the entire manuscript to ensure precision. In the revised discussion section, we have included more information (from secondary data and other sources) to complement our findings.

Specific comments:

Abstract
• The use of the term ‘urban form’ is awkward in the abstract without any explanation or description

*Authors’ response:*

we have added a brief dentition of urban form in the abstract.

Introduction

• First paragraph, page 2 – this paragraph is long and describes multiple concepts. Try to break up the paragraph into digestible chunks. For example, start a new paragraph when the author(s) start explaining adaptation.

• Pg 4 – the paper objectives need to be more clearly stated. Perhaps by breaking up the objectives into an overarching objective or research question followed by sub-questions. Do not bury this within a longer paragraph, make it stand out to highlight them. As of now they are spread throughout two paragraphs and not concisely stated.
• Please define Urban morphology more clearly for those who are unfamiliar.
• In general, I would shorten this section and put much of this text into the following theoretical background sections. The introduction could then be reduced to a short opening paragraph that states the problem, a second short paragraph highlighting research gaps, and then a third short paragraph that clearly and concisely stats the paper/project objectives. Then the background can be provided in the next sections.

*Authors’ response:*

Thank you for this suggestion. As recommended, we have shortened this section and made it concise. We added a brief definition of urban form and morphology to the main texts. Also, their detailed definition is available in the footnotes. The second to last paragraph of the introduction (Page 3) now discusses the paper’s main objective and key questions.

Contemporary discourses on urban resilience
• It is important to consider how resilience varies from adaptive capacity. They are often used interchangeably – so how do they differ when it comes to urban environments?

*Authors’ response:*

Thank you for this important feedback. We have done exactly this recommendation. The revised section now explains the relationship between resilience and adaptive capacity in the context of urban environments.

2.1 Urban form’s resilience and its measures
• A discussion of psychological resilience could be incorporated here when discusses Vale’s definition of urban resilience. While it developed separately from social-ecological resilience, there are certainly useful overlaps.
• Please define “urban form”
• Figure 1 is great – please refer to it more directly in the text.
• For each of the components in Figure 1 - please provide not only the theoretical explanations, but more concrete, real-world examples.

*Authors’ response:*

We thank the Reviewer for pointing out this issue. In fact, urban morphology (i.e., the physical dimension of urban design) is a key component of our work. We didn’t pay much attention to “psychological resilience” because it remains out of our scope. Yet, as advised by the respected Reviewer, we have discussed psychological resilience briefly to help our readers become aware of it and understand a broader definition of resilience.

We are glad that the reviewer finds the figure useful. We have added a few lines to explain it more. A definition of urban form is now available in the texts and endnotes.

2.2 A framework for Urban morphological resilience
• Figure 1 does not belong here. It belongs in the previous section where it is explained and mentioned first. The same is true in Table 1. I suggest moving it earlier.
• Table 1 could also use some reformatting. Try to make the columns with a lot of text wider.
• This section is generally not needed. I would add it to the section above.

*Authors’ response:*

We thank the reviewer for this suggestion. We have relocated Figure 1 and Table 1. And we have also shortened the table’s contents as much as possible since we discussed these contents in Section 2.1. Lastly, as advised by the reviewer, we have merged this section with the section that used to precede it.

Case Study and Methods
3.1 Geographic focus
• Statistics about the impacts would be helpful, such as measured and/or projected sea level rise/
• There are virtually no references in this section, please do cite sources.
• There is also no explanation for why this area is a good place to implement the proposed framework.

*Authors’ response:*

We agree with the reviewer and appreciate pointing out this lack. We revised the section according to the suggestions and cited more sources. We revised Fig 2 (location map) to include more data. Also, the section includes more evidence and statistics. We also discussed a few reasons for selecting Negril as a good case/place (please see the last paragraph of Section 3.1)

3.2 Data sources
• Subtitle 3.2.2 is not formatted correctly
• I would suggest moving 3.2.3 Study limitations to the end of the methods section. That is where it is most typically found

*Authors’ response:*

We revised the section according to the reviewer’s suggestion.

4. Measuring Long Bay’s resilience
• I am not sure the term ‘measuring’ is accurate. The following discussions do not make judgement statements or overall measurements, instead they discuss various aspects that relate to resilience.
• The figures are helpful, but hard to interpret at times
• It also might be useful to more successfully integrate the secondary data and GIS data. Showing maps of current infrastructure systems would be helpful and nicely complement the quotes.

*Authors’ response:*

We thank the Reviewer for this critical observation of using the term *‘measuring.’* We have reviewed the entire manuscript and revised the term.

We have added more explanations based on secondary data and other evidence to complement the quotes. We agree that Figures (4-7) are a bit abstract. The reason we kept them generic is to enable the readers to understand the main themes (without the contextual complexity of Negril).

5. Discussion and conclusions
• I suggest moving the quote that opens up Section 5 to the very beginning of the entire manuscript as it helps situate the research.
• The first paragraph is just one long run-on sentence. Please do revise. Also a figure that summarizes the main findings would be incredibly helpful.
• The author(s) could more closely tie the discussion/conclusions back to the topics discussed in the introduction.
• The authors should compare their findings to similar studies in other geographical areas.
• Figure 8 belongs in the results section since it is a result.

*Authors’ response:*

We thank Reviewer 1 for this suggestion. We moved the quote to the beginning of the manuscript. We revised this section and long sentences. In fact, Figure 8 presents a summary of our assessment. We added a few similar contexts (e.g., in India and USA) to the section so as to compare and complement our findings.

**Reviewer 2**

The paper is focused in a very important issue as climate resilience is. The author proposes a framework for asses this climate resilience based, essentially, on urban design. Although the six points discussed in the framework are important, I think that, obviously, they are not enough to face all the problems related to climate change. For this reason, I think that a clearer discussion about the scope will be welcomed.

*Authors’ response:*

We thank Reviewer 2 for appreciating our attempts. We have revised the introduction to make its scope and objective clear.

The literature review is wide but as the paper is mixing different areas, the final position remains unclear. In other words, is there other frameworks for assessing climate resilience? Could be better to focus on frameworks or in urban design?. I would appreciate a discussion on specific issue of the paper more than a general discussion on general models. In my case, the final idea of the discussion remains unclear. As the author said, I was expecting to limit the intersection between urban design and resilience, and to give specific actions for being done.

*Authors’ response:*

We thank the respected Reviewer 2 again for this constructive feedback. We revised the literature review section by omitting all irrelevant points and condensing the text to clarify and keep the focus on the main arguments. The revised manuscript now includes a few frameworks (for example, Allen 2001, Jabareen 2015, Quigley 2018, and Pickett’s 2013) that are also relevant to urban design but not directly associated with climatic variability. The revised sections also draw the theoretical foundation and application guidelines to integrate urban design and urban resilience in reference to other applications. We argue that by configuring the physical and special characteristics of urban environments, we can enhance climate resilience. We have added it to our introduction. We hope it makes the entire discussion clear, focused, and evidential.

In fact, the sixfold framework comes from six urban design concepts, but it is not enough discussed if is the main paper contribution. The emergence of the six concepts should be better explained and to specify why these six concepts.

*Authors’ response:*

We thank Reviewer 2 for pointing out these issues. We have tried to develop the framework as the main contribution of this paper. Indeed, there are several concepts that have been used in the urban design/planning literature and practiced over decades. However, we found that only six of them have the potential to link urban design (the focus of this paper) and climate resilience. In the revised section, we have emphasized this point.

Having frameworks helps to give order to assessing methods but, in general, the application of indicators and measures is the path. In this case, the author present indicators and variables but it is not possible to apply them to case study. Perhaps, storytelling needs to be rethought and strengthening the links between the framework and the application.

In any case, the work done with all the interviews is very valuable and should be appreciated by the politicians and public servants of the case study.
I was expecting new ideas and new ways to adapt to climate change in the case of increasing sea-level and it was not the case. In fact, economic and political barriers appear, as it is usual.

*Authors’ response:*

We thank the reviewer for identifying this shortcoming. Indeed, as the respected Reviewer 2 highlighted, the framework and its components are conceptual. And yes, certainly, not all of their indicators may be useful/applicable directly to a single case. Instead, these frameworks, ours included, should be carefully contextualized for the conditions and needs of each context. For example, in Negril’s case, we applied them to understand the baseline resilience based on the physical planning and application policies in the absence of quantitative data (e.g., projections). The secondary data and the expert opinions were used (from our interviews) to verify/validate our findings or vice versa. However, we focused more on the physical environment and urban design. We did not include the voice of politicians in this study because we focused on the experts. This is not to say that their input is not important, but to actually add it in future studies (for example, comparing experts’ and politicians’ views). For this study, we have added the lack of a socio-political lens to the limitations of this research and discuss it in Section 3.4 accordingly.

Results are very interesting for decision-making but the author is proposing actions that should be part of a resilience plan. For this reason, I consider that although the paper is interesting, it is unsuitable for publication in its present form. Nevertheless, the study shows sufficient potential to encourage the author to resubmit a revised version focusing on the main contribution to the academic community. In other case, the paper is nearer to a policy practice than an academic paper.

*Authors’ response:*

We thank you for the critical insight. In the revised conclusion we have highlighted the explicit contribution (theoretical and practical) of the paper to the academic community.

**Reviewer 3:**

The authors are presenting an interesting work; however, the current version of the manuscript has many flaws. Introduction (section 1) and Literature Review (section 2) are unnecessarily long. These sections need to be combined and reduced. Especially the concepts explained in the section 2 need to be summarized and reduced.

*Authors’ response:*

We thank Reviewer 3 for finding our work interesting. We condensed both sections (introduction and literature review) to ensure precision and brevity.

As for the concepts in Section 2, they represent the key components that contribute to the framework we have proposed. We have cut some of them and tried to reduce the section. We have added examples and different scholarly views that are relevant to make our arguments clear and logical.

No need to explain in detail when they have a summary table (Table 1) which presents the same information.

*Authors’ response:*

To avoid redundancy, we have shortened Table 1 as much as possible, which now presents only a summary of Section 2.1.

I prefer to rename the section 3 as Materials and Methods, and it need to be section 2.

Section 4 should be Results and Discussion and need to be changed to section 3.

*Authors’ response:*

We have revised them accordingly.

I also wonder why they do not have a standalone conclusion section. Therefore, I recommend major revisions to this paper so that the authors can have an opportunity to greatly improve their work that can be useful and readable.

*Authors’ response:*

We thank the reviewer for this suggestion. The revised manuscript now has a separate “standalone conclusion.” It discusses the paper’s contribution with more evidence and contexts similar to our case.
