# Supplementary material for: A sixfold urban design framework to assess climate resilience: Generative transformation in Negril, Jamaica
Source: PLoS One. 2023 Jun 23;18(6):e0287364. doi: 10.1371/journal.pone.0287364 (PMC10289356; doi:10.1371/journal.pone.0287364)
Supplement: Attachment — Submitted filename: Response to Reviewers.docx [file pone.0287364.s006.docx]

**Response to Reviewers (Round 2)**

PONE-D-21-17612R1

Title: A sixfold urban design framework to assess climate resilience: generative transformation in Negril, Jamaica

**Reviewer 1**

“Thank you for the thorough revision. This paper is much improved from the original draft. However, I still find that the Results and Discussion section needs major revisions. See my more detailed comments below. The clean version has some awkward spacing and punctuation – likely from the track changes revisions. Please do review the clean version to edit these small mistakes.”

*Authors’ response:*

We thank the respected reviewer for reviewing our manuscript again and for your valuable suggestions. We have carefully considered your suggestions to revise the manuscript thoroughly in this round and fix minor editorial issues (including typos, spacing and punctuation).

“Introduction – The first quote and paragraph are not well linked with the second paragraph. Try to write a transition sentence or something that links the two.”

*Authors’ response:*

We agree with you. We have revised the first two paragraphs to improve the link cohesively.

“Overall, the introduction is much improved. It is concise and well cited.

Contemporary discourses on urban resilience – This section is also much improved. It is well organized, and provides a thorough definition of each concept.

Methods – Clear and concise.”

*Authors’ response:*

Thank you so much for valuing our efforts.

“Conclusion – some of this could be moved to the results and discussion section as the overall summary of the results.

*Authors’ response:*

We really appreciate your suggestion. Accordingly, we revised the Results and Discussion (the last part) and Conclusions section significantly.

I do like how the authors have organized the last paragraph by the theoretical, empirical and practice- wise contributions – but I would recommend breaking up each of these into its own stand alone paragraph. That would help each contribution stand out.”

*Authors’ response:*

We are glad to know that our discussion on theoretical, empirical and practice-wise contributions has become effective. In this revised version, we have separated each contribution.

**Reviewer 2**

“It is more a theoretical article than an applied one. For this reason, the data doesn't support all conclusions. It is not so nice for reading, in some parts, you need two read twice or three times for understanding the sense of the content.

The article has improved but section 2 needs to better clarify what is the final goal. It is difficult to understand for people that is not coming from urban side. Storytelling is not well designed and some gaps remain in this section.”

*Authors’ response:*

We thank the respected reviewer for highlighting the key notion of our paper, i.e., toward establishing the theoretical link among interdisciplinary components in responding to climatic risks.

We revised our manuscript thoroughly to improve its readability. In doing so, we have shortened sentences to simplify our arguments in many sections. We also revised Section 2 (i.e., Contemporary discourses on urban resilience), where we developed the foundation of our theoretical framework – one of the major goals of this paper (as discussed on Page 4 and in our abstract).

We consider your comments seriously. In this revision, we have revised all sections to be suitable for a general audience, particularly those who are not from the urban side. We defined “urban design” in Introduction for such readers. Also, a new endnote (which describes urban design more along with its origin and scope) has been added for general readers who are less/not familiar with urban design but are interested in the field. We believe that the three endnotes (on urban design, urban form, and urban morphology) will help such readers.

“The title of 2.1 is not linked to the text, I’m not able to find measures of urban form’s resilience. In fact, in table 1 you are using “concepts” word and no measures. Title is not corresponding to the content of this point. In case you are using measures devoted to ways of measurement, it is necessary to clarify because, for me, the word measures are measures for action to be taken by someone.”

*Authors’ response:*

We thank you for pointing this out. We completely agree with you. We changed the title of Section 2.1 to better link to the contents of the section as well as Table 1.

“In page 10, you need to change 3.1 for 2.1 Geographic focus because it is under 2. Materials and Methods section.”

*Authors’ response:*

We have corrected all numbers of the titles and subtitles and their sequences.

“Concerning 3.4 Study limitations and advantages, my point is it is about data or about the study. In this case, you need to move the section to the end.”

*Authors’ response:*

We thank the reviewer for sharing the critical insight. We have moved this section to conclusions.

“Results seems to derive of the framework application but it remains confusing. It could be better to stress this link in order to give more sense to the proposed framework.”

*Authors’ response:*

We thank the reviewer for this suggestion. Throughout the manuscript, we referred to many cases and applications of other areas to help our readers understand the link. We revised our conclusions and added a new Section 4.7 (Negril’s resilience and a path toward generative transformation) to clarify the link (both theoretical and empirical) that is the key objective of the proposed framework.

“I’m not used to include new references in conclusions. The discussion has been made previously.”

*Authors’ response:*

In this revision, we removed all references from the conclusions.

**Reviewer 3:**

“I thank the authors for substantially improving their manuscript adhering to the reviewers’ comments. However, I suggest authors to go through the entire manuscript and check for typos and minor spell/grammar mistakes. It is also better to double check all the citations and references. In some places, fonts were different as well.”

*Authors’ response:*

We thank the respected reviewer for considering our last revision effective and improved. At this round, we have double-checked and addressed all minor editorial mistakes and citation issues to improve the quality of our paper further.

“Title of the section 4 needs to be just Results and Discussion and the rest in the title can be removed.”

*Authors’ response:*

We revised the titles as advised. Thank you.

“Conclusion section is very long. I would suggest removing the citations and referencing to the previous work in the conclusions so they can cut it down to 2-3 paragraphs. Conclusions should highlight the key findings of the study with limitations and recommendations for future work.”

*Authors’ response:*

Thank you for this intellectual advice. We have revised our conclusions significantly. We condensed the section, highlighting our key findings and contribution with limitations and recommendations. We have also removed all citations.
