# Supplementary material for: A sixfold urban design framework to assess climate resilience: Generative transformation in Negril, Jamaica
Source: PLoS One. 2023 Jun 23;18(6):e0287364. doi: 10.1371/journal.pone.0287364 (PMC10289356; doi:10.1371/journal.pone.0287364)
Supplement: Attachment — Submitted filename: Response to Reviewers.docx [file pone.0287364.s007.docx]

**Response to Reviewers (Round 3)**

PONE-D-21-17612R2

Title: A sixfold urban design framework to assess climate resilience: generative transformation in Negril, Jamaica

**Reviewer 1**

The authors have not addressed all of my revisions and concerns. They did not respond to the following and important suggestion from the last round of revisions:

"Results and Discussion – This section is still lacking much of a ‘discussion’. It serves mostly to summarize the results, but does not place it into the larger context nor compare results with other research. There are very few citations. Additionally, there is no overarching section where the author(s) directly speak to how the results answer their research questions. This is important to include. It is also important to generalize these results. How could this framework be used and applied elsewhere? How do the results compare with other similar studies? This type of discussion is lacking, and this section is mostly a results section."

They did add one section, but the rest of the section was not revised according to my suggestions.

*Authors’ response:*

We considered (and are considering) all reviewers’ comments seriously to take every opportunity to improve our manuscript. In fact, we addressed the concern of the respected Reviewer-1 and revised our manuscript accordingly. We also wrote how we made the revisions in our response letter. However, unfortunately, our responses to this particular section have somehow been missed/deleted while handling the file. It was a mistake, but it happened unintentionally. Please accept our sincere apologies.

I would like to add the following responses that were missed in our previous letter:

“In this revision, based on our data, we discussed our findings in Sections 4.1 to 4.6. Each of these sections assesses the study area based on our proposed six concepts while connecting the field of urban design (and planning) and climate change resilience. Some of our concepts have long been discussed in the literature. For example, *Ecological Sensitivity* has existed in “urban ecology,” “design with nature,” and “landscape ecological urbanism” for a while, and their renewed interest in “eco-based adaptations” and “nature-based solutions” is emerging given the climate crisis. However, other concepts are new to the field (e.g., *Redundancy* and *Indeterminacy*), and their applications are rare in the context of climate change. Section 2.1 discusses it and presents a review of international cases (albeit they don’t relate to climate change) to clarify the six concepts and their similar applications worldwide. We didn’t repeat them in Sections 4.1 to 4.6. Instead, these sections shed more light on the application of each concept to the Long Bay area so as to understand and assess its resilience through its spatial layout and physical configuration–an objective of this paper. To generalize our results, we have added a new section (Section 4.7: Negril’s resilience and a path toward generative transformation) to explain our results in a broader context while comparing them with other studies.”

In this round, we thank the respected reviewer again for reading and sharing his/her/their comments with us. We take another opportunity to carefully review the newly added Section 4.7 (marked in yellow) and revise the entire section again. The section now includes a few examples from the Caribbean and similar contexts to generalize and compare our results. It helps readers understand our concepts better and their applications beyond Negril. We believe the contents of this section also help readers understand the paper’s contribution (theoretical, empirical, and practice-wise), as we discussed in the following Conclusions sections.
